# ‘A heart within, a God overhead’: the making of nuclear medicine in postcolonial India, 1947–1985

**DOI:** 10.1017/mdh.2026.10076

**Published:** 2026-10

**Authors:** Kapil Patil

**Affiliations:** Shiv Nadar Institution of Eminence, India

**Keywords:** Nuclear Medicine, Radiosiotopes, Atoms for Peace, India, Postcolonial Science, Technoscience

## Abstract

The onset of the atomic age in India embodied a unique postcolonial ambition to harness atomic energy for peaceful purposes. Within this vision, nuclear medicine became a key instrument to modernise the country’s colonial healthcare infrastructure. For India’s nascent community of radiologists, radioisotopes offered a promising frontier in clinical research and therapeutic applications. Yet the development of nuclear medicine was far from a straightforward extension of atomic research into clinical practice. Instead, it took shape within a centralised framework that concentrated technological resources and expertise in a small number of metropolitan centres. The centralisation of nuclear medicine practice was not merely a response to the resource constraints of a postcolonial economy. It also reflected a technopolitical project that linked the modernisation of public healthcare to expanding atomic energy infrastructure. Situating the emergence of nuclear medicine within the intertwined histories of radiology, cancer care, and atomic energy research, the paper examines the postcolonial aspiration to harness advanced medical specialities through the atomic enterprise. In doing so, it unpacks the technopolitics of nuclear medicine, highlighting the politico-epistemic negotiations that shaped its institutionalisation in India.

## Introduction

The history of nuclear techno-science in postcolonial India has attracted considerable scholarly attention for its multifaceted character and motivations. A growing scholarly oeuvre has shown how India’s atomic energy program emerged through the interaction of postcolonial nation-building, the pursuit of international prestige, and efforts to overcome the legacies of colonial underdevelopment.^1^ In particular, Jahnavi Phalkey has demonstrated how the creation of India’s atomic infrastructure became intertwined with the construction of state authority and its quest for scientific modernity. Itty Abraham has similarly situated the development of India’s nuclear program within broader processes of postcolonial state formation, secrecy, and global power relations.^2^ Despite this rich scholarship, the genesis of the nuclear medicine field in India presents a striking though underexplored manifestation of its techno-scientific imaginations.

Catalysed by Cold War initiatives promoting the peaceful uses of atomic energy, Indian policymakers, atomic energy leaders, and radiologists viewed radioisotope-based diagnosis and therapy as instruments of both medical modernisation and national development.^3^ For the postcolonial state, nuclear medicine not only promised to demonstrate the beneficial applications of atomic energy but also to modernise the country’s archaic healthcare system. For radiologists, it offered an opportunity to overcome what many perceived as a chronic ‘technological lag’ and align Indian clinical practice with international standards.^4^ Importantly, for India’s postcolonial polity, nuclear medicine, as ‘a peaceful’ atomic energy tool, was diplomatically significant.

As Prime Minister Jawaharlal Nehru remarked at the inauguration of the ‘Atoms for Health’ exhibition in 1961, the challenge of the atomic age was to transform atomic energy from an instrument of destruction into a force for human welfare, i.e., of ‘turning swords into ploughshares’.^5^ While Nehru’s advocacy of harnessing atomic energy for beneficial ends was crucial in its global context, his affirmation of placing nuclear medicine and cancer research within the ambit of India’s atomic energy program was not without its share of contestation.^6^ Also, within India’s healthcare system, burdened by infectious diseases, malnutrition, and inadequate primary care, a resource-intensive speciality such as nuclear medicine would raise concerns about cost, technological capacity, and equitable access.

In this backdrop, the paper examines how and why nuclear medicine emerged under the aegis of India’s atomic energy establishment in the decades following independence. It mainly contends that nuclear medicine was neither a straightforward transfer of Western medical technology nor a misplaced project of modernisation for a postcolonial state. Rather, its emergence within India’s atomic energy programme was fundamentally technopolitical, revealing a deeper intertwining of political and institutional authorities.^7^

Drawing on Gabrielle Hecht’s framework of technopolitics, the article seeks to illuminate three interrelated logics that drove the development of nuclear medicine in postcolonial India: first, its role in demonstrating the broader social utility of atomic energy while extending the remit of the atomic energy establishment into the healthcare domain; second, its construction as an instrument of healthcare modernisation through centralisation of expertise and infrastructure within specialised institutions; and third its consolidation through Cold War scientific cooperation, which embedded India within transnational circuits of scientific knowledge, materials, and expertise. Examining these interconnected dynamics reveals the technopolitical processes underlying the genesis of the nuclear medicine field.^8^ The establishment of the Radiation Medicine Centre (RMC) at Bombay’s Tata Memorial Hospital best exemplifies the development of a highly centralised, state-mediated framework for nuclear medicine.

The concentration of expertise, infrastructure, and resources within a small number of specialised institutions, rather than diffusing them across the healthcare system, was seen as consistent with international norms governing advanced nuclear medicine specialisation and as accommodating the structural limitations of India’s medical-industrial capacity. Moreover, its clustering around atomic energy created an important institutional nucleus for advancing an integrated biological and medical research program at the atomic energy establishment. To understand the processes underlying nuclear medicine’s organisation in India, the paper situates it within the broader histories of radiology, cancer care, and the development of India’s atomic energy infrastructure.^9^ This historical anchoring is crucial to delineate a distinct postcolonial context within which India’s nuclear medicine practice emerged and evolved.

Moreover, it is essential to move the historiography of radiology and nuclear medicine science beyond the Eurocentric scope and to highlight diverse themes in the history of medicine in the Global South. These mainly include concerns such as the politics of postcolonial health and centre–periphery relations in the production of scientific knowledge. In *The Rise of Western Medicine in India*, David Arnold shows how Western medicine advanced rapidly under colonial rule, even if its authority remained contested, negotiated, and unevenly institutionalised. Pratik Chakrabarti’s *Medicine and Empire, 1600–1960*, by contrast, emphasises the entangled, circulatory, and co-produced character of medical knowledge within imperial formations, challenging the notion of linear knowledge transfer from metropole to colony. Robert D. Smith likewise dwells on the history of cancer care, arguing that the use of radium therapy in India became legitimised amidst the intertwining of philanthropic and techno-nationalist factors.^10^

While these scholarly works are crucial for understanding the colonial genealogy of healthcare regimes, there is relatively little attention to nuclear medicine as a site through which atomic energy entered the public health domain. Building on these contributions, the paper maps the emergence of India’s nuclear medicine program, including its prehistory within the practice and institutional norms of radiology and *radiotherapy.* ^11^ Drawing on a range of archival sources, such as professional journals, parliamentary debates, and international assistance records, the paper illuminates a context-specific trajectory of India’s nuclear medicine enterprise, shaped by specific actors and institutional networks. Unpacking this context is crucial to appreciating the diverse institutional trajectories of nuclear medicine globally.^12^ In turn, the paper charts the medical history of nuclear science, attending to its unique institutional lives in India’s postcolonial settings.

### Radiology (and radiotherapy) in a developing world

The genesis of radiology and its allied sciences has been a vital area of historical inquiry because of their complex origins and trajectories. While the extant historiography of radiology has largely remained Eurocentric, reflecting its Western origins, in the developing world, it spread mainly through colonial encounters, imperial rule, and wartime efforts. As Pratik Chakrabarti argues, European powers strategically mobilised modern medical advances to support empire-building projects.^13^ The establishment of one of the oldest X-ray facilities at the Madras General Hospital (now Chennai) in 1900 by British military surgeons was part and parcel of colonial administration in India.^14^ The end of colonial rule in August 1947 marked a critical juncture, enabling the postcolonial Indian state to pursue an autonomous programme of scientific and technological development.

Owing to longstanding colonial neglect, India’s healthcare system at the time of its freedom was evidently in a precarious condition, with an alarmingly low number of physicians per capita.^15^ While modern medical facilities were available almost entirely in urban centres, over 80 per cent of India’s population resided in rural areas. Radiological services were similarly inadequate for the country’s size and population, with an acute shortage of equipment and qualified radiologists. At the dedicated cancer hospitals of Bombay, Madras, and Calcutta, radiologists lamented their inability to keep pace with scientific and technological advances in their field. Upon independence, Indian radiologists thus called for an immediate expansion of radiological services by addressing the shortage of trained personnel and affordable technological resources, as well as creating capacities for advanced teaching and research.

The revival of the Indian Radiological Association (IRA) in 1946, in this backdrop, marked an important moment for the progress of radiology in the country.^16^ The IRA not only marked the formation of India’s radiological community but also, from its very inception, began sensitising Indian policymakers to the challenges of the radiology field and to shape its institutional and regulatory contours. Proclaiming with pride that ‘without the help of radiology, medicine would have been in a state of glorified quackery’, the IRA viewed itself as a key stakeholder in building a modern healthcare system in the country and set itself out to raise standards of Indian radiology.^17^ In the decade following its revival, the IRA particularly focused on three interrelated issues: the establishment of a domestic X-ray electro-medical industry; the regulation of radiological training and practice; and the institutional organisation of radiotherapy in India.

At its first annual congress, held in Madras in 1946, the IRA adopted an important resolution on the development of the X-ray and electromedical industries.^18^ Having suffered acute shortages of films and chemicals during the war, the association promptly communicated its resolution to the central and provincial governments, urging them to prioritise the development of a domestic radiological industry. Such an industry was essential to ensuring the local availability of affordable radiological materials and extending radiological diagnosis to the masses.^19^ The IRA continued to press Indian policymakers to establish a domestic radiological industry in subsequent years, recognising that building domestic industrial capabilities in this field would be a long-drawn process.^20^

The association also focused on developing radiological teaching and research capacity in the country. In February 1947, it launched the *Indian Journal of Radiology*, which soon became a platform for deliberating on key epistemic and regulatory issues.^21^ In 1950, the IRA submitted its recommendations to the government, advocating legislation to govern Indian radiological practice.^22^ As the use of radiation in diagnosis and therapy began to increase rapidly post-1947, the question of who should be permitted to practice radiology became paramount for the association, especially amid reports of unchecked radiology practice by medical and non-medical practitioners lacking specialised training but who could simply afford to purchase X-ray equipment. The IRA, on this matter, opined that only ‘fully qualified radiologists’ should be authorised to use X-ray equipment and radioactive substances.^23^ The IRA’s stance, however, met with opposition from another influential medical body, the Indian Medical Association (IMA), whose members argued that such strict qualifications infringed upon the ‘right’ of general practitioners to use X-ray machines.^24^ Following a prolonged disagreement, the IMA eventually endorsed the IRA’s concerns regarding radiological malpractice.^25^

Similarly, for the training of radiologists, the IRA recommended a compulsory two-year postgraduate program, complete with defined eligibility criteria and a structured curriculum. Over time, the debate over the organisation of radiological training and practice intensified, prompting the IRA to periodically review its stance. Two interrelated concerns shaped these discussions. First, in the face of ‘reportedly’ higher incidence of cancer and pulmonary tuberculosis, the association recognised separate roles of radiodiagnostic and radiotherapy services in the country.^26^ Although there was no established parity in cancer incidence rates between India and the West, cancer specialists like V.R. Khanolkar from Bombay appealed for more studies on the subject.^27^ Second, association members also pointed to the separation of diagnostic and therapeutic radiology into distinct specialities in many advanced countries. However, since the overall number of teachers and institutions in India was quite small, the IRA initially advocated a two-year ‘combined though intensive course of studies’.^28^ Eventually, it came around to recognise the separation between diagnostic and therapeutic radiology in both training and clinical practice.

In response to the mounting burden of tuberculosis and the need for mass radiography, the association called for rapid expansion of radiodiagnostic facilities, especially in smaller and remote areas. In this regard, it also called for ‘progressively extending’ radiological teaching centres from cities to smaller towns and villages.^29^ In the case of radiotherapy, though, the IRA acknowledged its specialised character and role in cancer treatment.^30^ At the IRA’s 1948 session held in Bombay, Mr Jivaraj Mehta, the Director General of Health Services, called for coordinated cancer diagnosis and treatment across institutions in public and private domains. For this purpose, he proposed establishing Chairs of Radiology at medical colleges where surgeons, radiologists, physicists, and biochemists would collaborate in research and clinical practice.^31^ At this stage, radium therapy was already prevalent alongside surgical procedures at India’s prominent metropolitan hospitals, notably the Chittaranjan Cancer Hospital, Calcutta; the Bernard Institute, Madras; and Bombay’s Tata Memorial Hospital (TMH).^32^

For the IRA members, radiotherapy (comprising radium and deep X-rays) was vital as both ‘curative and palliative therapy’. Yet, over time, they also began expressing caution about any exaggerated claims regarding radiation treatment. K. P. Mody, a radiologist at TMH, for instance, warned against a popular belief that the ‘terrific release of energy from the break-up of an atom’ could simply be used to destroy malignant tissues. Also, on the broader question of whether ‘atomic energy was the answer to the great problem’ (of cancer), the IRA adopted a position that balanced the perceived benefits and limitations of ionising radiation.^33^ Acknowledging that the radiation techniques had reached ‘a certain threshold of efficiency’, they emphasised an ‘early diagnosis’ and ‘careful case selection’ to ensure effective treatment and cure.

This position, along with the qualifications attached to the therapeutic use of radiation, was notable for its pragmatism. Amid increasing public awareness of radium controversies and the dangers of excess radiation exposure, it served to resist the tendency to exceptionalise radiation as a uniquely effective cure for cancer. Emerging technologies such as radioisotopes were therefore unlikely to be treated differently, despite radiologists’ enthusiasm for their widely publicised clinical and research potential. In doing so, the IRA members, like Mody, offered a more measured understanding to temper the enthusiasm around radiation as a panacea for cancer and called for raising public awareness on early detection and treatment of various malignant diseases. The establishment of the Indian Cancer Society in Bombay in 1951 marked a step in that direction.^34^

At this stage, the radiological community also began debating the organisational structure of radiotherapy in India. Successive IRA leaders, on this issue, referred to the Western practice of nucleating experts and resources in centralised institutions to achieve effective results. Rather than dispersing limited funds across multiple under-resourced institutions, the IRA members favoured establishing one or two specialised radiotherapy centres for training and research that would account for the resource constraints of a postcolonial economy. Criticising the prevailing ‘old custom’ whereby Roentgen (X-ray) treatment was associated with radiodiagnosis and radium with surgery, N. B. Roy, the sixth IRA President, held that real progress was not possible until the two specialities were fused into a separate and distinct speciality of radiotherapy.^35^ Moreover, invoking the ideal of ‘Heart within and God Overhead’ and likening physicians to soldiers on the battlefield, Roy made a passionate plea to establish a centralised radiotherapy institution equipped with the resources and expertise needed to lead the fight against cancer.^36^

Echoing Roy’s vision, the association’s General Secretary, R. F. Sethna, likewise emphasised that radiotherapy – encompassing X-rays, radium, and radioisotopes – required specialised teams and a range of auxiliary services. Noting that such infrastructure can be effectively organised only within centralised institutions, he supported the argument for centralising radiotherapy services across major cities.^37^ Given the limited career opportunities for advancement in radiotherapy, Sethna recommended that only committed young people be sent abroad for training in modern methods, including the use of radioisotopes.^38^ For these leading Indian radiologists, the creation of a centralised radiotherapy institution was reasonable in light of prevailing Western practices and the imperatives of resource optimisation. In a postcolonial setting, however, the organisation of radiotherapy became embedded in an important technopolitical scheme – i.e., to rationalise atomic energy development for its social benefits and to extend its institutional remit to healthcare modernisation. The following section traces how this technopolitical vision shaped the emergence of India’s nuclear medicine programme within the country’s atomic energy apparatus.

### The technopolitics of nuclear medicine

By the turn of the 1950s, the extensive publicity surrounding radioisotopes – particularly in the United States – had not escaped the attention of Indian radiologists. Radioisotopes were among the founding discoveries of the atomic age, gaining credence for their utility in medicine, agriculture, industry, and research.^39^ Moreover, in the aftermath of the Hiroshima and Nagasaki bombings, they became politically significant in the transformation of the destructive image of atomic energy in popular perceptions and to project its beneficial side.^40^ Celebrated as ‘new laboratory spies’ and ‘the most useful research tools since the invention of the microscope in the seventeenth century’ for the study of biology, the U.S. Atomic Energy Commission (AEC) sought to distribute radioisotopes to the European allies as part of its broader programme of postwar scientific cooperation and reconstruction.^41^

Radioisotopes such as Iodine-131, Cobalt-60, and Technetium-99m, in particular, transformed the field of radiotherapy, facilitating the emergence of new disciplines, including nuclear medicine.^42^ Conscious of the fact that India had made ‘very little contribution to the advancement of the world’s knowledge of radiology’, the Indian radiologists were eager to embrace these new tools and explore their diagnostic and therapeutic potential. This opportunity emerged, albeit on a limited scale, with the British Atomic Research Establishment at Harwell supplying smaller shipments of radioisotopes in the early 1950s.^43^ Following the U.S. lead, the British government also began using radioisotopes as political tools to strengthen ties with Commonwealth countries. The allied policy of replacing radium with radioisotopes and distributing them at minimal cost particularly drove their widespread use in therapy.^44^

The use of isotopes from Harwell for cancer treatment at Calcutta’s Chittaranjan Cancer Hospital was even reported in the national press.^45^ TMH and the Bernard Institute in Madras, too, received radioisotopes from Harwell. Yet Darab Jehangir Jussawalla, a surgical oncologist at the TMH, noted that without governmental assistance in establishing a dedicated radioisotope laboratory, India risked lagging ‘by about 10–15 years’ in its work.^46^ Echoing a similar concern, ANK Menon, IRA President in 1960, lamented that nuclear medicine in India trailed behind more advanced countries. On behalf of the association, he proposed establishing an Indian Institute of Nuclear Studies modelled on the United States’ Oak Ridge National Laboratory to train scientists in medicine, agriculture, and industry.^47^ Menon’s proposal built upon earlier calls by N. B. Roy for a limited number of ‘centralised radiotherapy institutes’ with attached peripheral cancer detection clinics.^48^ Together, these proposals reflected a growing conviction among Indian radiologists that the application of radioisotopes required a specialised institutional framework. In the postcolonial context, however, the creation of such an institutional structure became entangled with a broader technopolitical project of consolidating scientific authority around atomic energy.^49^

Despite its status as a key professional body, the IRA’s role remained primarily advisory. As such, radiologists and medical practitioners keen on using radioisotopes looked to the state for support of scientific research and advancement. As radioisotope techniques expanded globally, Indian radiologists linked their professional advancement to the progress of their country’s atomic energy program. Homi Jehangir Bhabha, the architect of India’s atomic energy program, became a pivotal figure in this alignment.^50^ The radiologists revered Bhabha for his international stature and bestowed upon him an honorary membership of the association in 1952 and the Sir Jagadish Memorial Medal in 1959.^51^ Having twice addressed its annual meetings, Bhabha was well aware of the challenges of Indian radiology and promised support for the supply of radioisotopes and the development of an X-ray electromedical industry.

As Janhavi Phalkey argued, in postcolonial India, the survival and expansion of scientific projects depended crucially on navigating state bureaucracies.^52^ In this respect, Bhabha embodied what postcolonial historians have described as the rise of ‘scientocracy’, a technocratic elite that exercised considerable influence over the state’s science policy and the allocation of public resources.^53^ Having effectively established atomic energy as a state concern, Bhabha also sought to extend its institutional remit into allied scientific fields, including medicine, biology, and space research. Following the first Geneva Conference in 1955, the applications of radioisotopes gained global momentum. In India, the launch of the first research reactor, Apsara, on 4th August 1956, enabled the production of certain radioisotopes in smaller quantities. Also, in Delhi, a military physician, Lieutenant Colonel Santosh Kumar Mazumdar, trained at London’s Hammersmith Hospital, established a radioisotope unit at New Delhi’s Safdarjung Hospital to treat thyroid disorders among both civilian and military personnel.^54^

At this juncture, Cold War geopolitics came to exert a unique influence on the development of nuclear medicine in India. The Eisenhower administration’s ‘Atoms for Peace’ initiative, which sought to win the allies for the U.S. in the developing world, led to India becoming a major beneficiary of U.S. atomic energy aid.^55^ Bhabha’s international scientific stature, reinforced by his presidency of the Geneva Conference, together with India’s growing strategic importance in the Cold War, facilitated Canadian assistance under the Colombo Plan to construct a high-capacity research reactor in India.^56^ Canada had earlier donated a few Cobalt teletherapy units to Indian hospitals. However, its decision to supply India with an NRX-type (National Research Experimental) research reactor, similar to the one at Chalk River, proved far more consequential in shaping Bhabha’s plans to develop nuclear medicine.^57^ The launch of the reactor in June 1960 positioned India among the first developing countries to produce radioisotopes on an industrial scale, thereby setting the stage for extensive use of radioisotopes in Indian medicine.

The Atomic Energy Department began to actively showcase the medical and other applications of radioisotopes at various exhibitions, including the World Agriculture Fair (WAF) organised in New Delhi in December 1959. At the Fair, the DAE had set up a makeshift radionuclide laboratory, distributing samples such as Sodium-24 and colloidal gold (Au-198) to Mazumdar’s unit at Safdarjung Hospital and to Vallabhbhai Patel Chest Institute.^58^ In February 1961, Mazumdar organised an exhibition titled ‘Atoms for Health’ in New Delhi. The event was notable for being inaugurated by Prime Minister Nehru himself. Using the occasion to affirm his government’s commitment to using atomic energy for ‘peaceful’ ends, Nehru urged, ‘it has become increasingly important that we should lay stress on this aspect of atomic energy which is beneficial to humanity’.^59^ His strong emphasis on harnessing atomic energy for peaceful purposes conveyed a subtle diplomatic message to Western powers, especially on the issue of atomic weapons tests and radioactive fallout. At the same time, Nehru’s endorsement of incorporating nuclear medicine and cancer research under the aegis of India’s atomic energy program reflected his domestic priorities and the technopolitics of the field.^60^

The considerations of research efficiency and impact only partially explain this decision. More broadly, it reveals a technopolitical feature whereby the state consciously expanded its atomic energy portfolio by incorporating nuclear medicine and cancer research. The debate over the organisation of atomic energy research reveals the tensions underlying this process. At the parliamentary session, Naushir Bharucha, a parliamentarian from the East Khandesh constituency, questioned Nehru about the policy of concentrating diverse research activities–physics, chemistry, engineering, and biological and medical research – all within the DAE.^61^ Arguing that the department was ‘cluttered’ with too many activities, Bharucha called for their disaggregation to ‘benefit the progress of atomic energy research’.^62^ Referring to DAE’s Biology and Medical Group, Bharucha held that such activities could be ‘safely transferred either to the Universities or the Medical Research Centres’, on the grounds that not all radiation-related research ought to fall within the remit of the atomic energy establishment.^63^

Nehru, however, rejected Bharucha’s contention as a ‘wrong approach’, maintaining that only an integrated and concentrated structure could yield effective results.^64^ Noting that certain aspects of radiobiological research can be conducted in hospitals, the fundamental work must be carried out in a ‘much more concentrated and integrated way to produce results’ (for cancer cure). Nehru’s response thus reaffirmed the role of Bhabha’s Department of Atomic Energy (DAE) as a principal institution undertaking nuclear medicine work. Nehru’s reference to a ‘cancer cure’ was similarly important in this context. IRA members like K. P. Mody had already cautioned against exaggerated claims regarding radiation therapy. Nevertheless, as historian Soraya Boudia contends, the promise of a cancer cure had emerged as ‘salient predilection’ among nuclear institutions worldwide.^65^ Indian policymaking proved no exception in this regard, and the linkage between atomic research and the prospect of cancer cure drove the expansion of the DAE’s biomedical program.

Nehru’s support proved equally decisive when Bhabha proposed transferring the Tata Memorial Hospital (TMH) from the Ministry of Health to the DAE and expanding its medical research program. Dissatisfied with the Health Ministry’s limited support for cancer and isotope work, TMH physicians and Tata Trust members had persuaded Bhabha to bring the hospital under the aegis of DAE.^66^ Developments elsewhere, especially in the U.S., reinforced this proposal. The establishment of dedicated clinical cancer research hospitals at Argonne and Oak Ridge National Laboratories by the U.S. AEC provided a useful precedent for Bhabha to initiate the hospital’s takeover by the DAE.^67^ Prime Minister’s note to Cabinet Secretary Vishnu Sahay indeed acknowledged the ‘great importance’ Bhabha had attached to the medical uses of atomic energy.^68^ Supporting his plea for the transfer of TMH, Nehru not only endorsed Bhabha’s contention that nuclear medicine would ‘set a pace in the medical field in India and indeed elsewhere’ but also expressed confidence that closer integration between atomic research and medicine might lead to ‘outstanding research work’.^69^

This political endorsement of the DAE’s expanding role in biomedical research was crucial. The transfer of TMH was not merely administrative in this respect. It established the DAE’s leadership in radioisotope production, nuclear medicine, and cancer research and care. Backed by Nehru’s political authority, Bhabha was able to consolidate a technopolitical arrangement in which TMH became the principal place of a centralised radiotherapy and nuclear medicine infrastructure, which many IRA members had long envisioned. Moreover, to integrate nuclear medicine into the TMH’s protocol and culture, Bhabha planned a separate unit, the ‘Radiation Medicine Centre’ (RMC), within the TMH in 1963. For Bhabha, RMC was going to be more than just a centralised research institution; it embodied an ambitious sociotechnical imaginary of healthcare modernisation. Bhabha reportedly envisioned RMC as a ‘mini–Mayo Clinic’, staffed by internationally trained physicians and equipped with advanced technologies, reflecting the aspiration to foster a world-class medical practice in India.^70^

Bhabha’s choice of the term *radiation medicine*, rather than ‘nuclear medicine’, for the centre was also significant, enabling the DAE to emphasise the full spectrum of radiation-based diagnosis and therapy. On this issue, Bhabha apparently consulted several international experts, including John Lawrence of UC Berkeley and Richard Taylor of the UK’s Radiochemical Centre at Amersham. On the issue of TMH transfer, Lawrence readily endorsed the TMH’s liaison with DAE’s medical program, calling it ‘a unique opportunity … with immense possibilities in research, diagnosis, and therapy’.^71^ Lawrence’s characterisation of Bhabha as ‘friendly to the United States and to me’ underscored the personal and diplomatic rapport between Indian and American experts in the late 1950s. Lawrence readily acted on Bhabha’s request for American expertise to help establish the DAE’s radioisotope program. The USAEC reportedly provided a US$120,000 grant to support the processing and nationwide distribution of radioisotopes in India. Also, the diplomatic agreement between the two countries enabled Indian medical personnel to receive training in the United States.^72^ Founding RMC specialists, including Ramanik Ganatra, S.M. Sharma, and Homai DaCosta, were all trained at U.S. institutions.^73^

The International Atomic Energy Agency (IAEA) and the World Health Organisation (WHO) equally supported capacity development in India through on-site training and the deputation of international experts to the RMC. As DaCosta recalled, there was an ‘uninterrupted flow of international consultants’ over the next two decades.^74^ The development of nuclear medicine, therefore, also entailed what Maria Rentetzi and Kenji Ito refer to as the convergence of nuclear scientific and diplomatic networks during the Cold War.^75^ Established on 3 September 1963 at TMH, the RMC began offering nuclear diagnostic and therapeutic remedies for vitamin malabsorption and thyroid disorders. With the standardisation of thyroid uptake tests in accordance with the IAEA reference protocols, the RMC gradually shifted its focus to common diseases in India, including liver diseases, anaemia, childhood steatorrhoea, tropical sprue, and protein-calorie malnutrition.^76^ Similarly, the experts at the Institute of Nuclear Medicine and Allied Sciences (INMAS) in New Delhi, founded by Santosh Kumar Mazumdar and Daulat Singh Kothari, developed the improved protocols for Iodine-131 to manage thyrotoxicosis in the ‘goitrous belt’ of northern India.^77^ Following nearly two decades of deliberations and advocacy, nuclear medicine took institutional shape in India’s metropolitan centres by the late 1960s, marking the convergence of professional ambitions, technocratic state-building, and Cold War internationalism.^78^

### From long teething troubles to a gradual expansion

Having institutionalised it within a centralised framework, nuclear medicine practice nonetheless encountered challenges in its initial decade due to India’s late industrialisation and limited technological capabilities. Subsequently, policy reviews and assessments began to emerge among policymakers and other stakeholders regarding nuclear medicine’s scope, utility, and efficacy. Various reports from the 1960–70s offer insights into the nature of technological challenges and policy priorities concerning nuclear medicine practice at TMH and elsewhere in India. Among these, the challenges of technology and infrastructure were particularly acute in the initial decade, much as they had been for radiology practitioners, affecting both its implementation and social reach. Just as radiologists had lamented the absence of a robust domestic radiological industry, nuclear medicine practitioners too faced challenges with instrumentation, including outdated, slow rectilinear scanners in the following decades.^79^

Although the DAE’s radiopharmaceutical program was steadily advancing thanks to reactors like CIRUS, the slow speed of rectilinear scanners was a serious constraint. The slow-paced imaging device permitted only Iodine-131–labelled radiopharmaceuticals for liver and thyroid scans, thereby restricting both the scope of diagnostic procedures and the number of patients who could be examined. Reports submitted by two visiting international experts to RMC particularly underlined these technological challenges. At RMC’s request, the International Atomic Energy Agency (IAEA) deputed two U.S. experts, Nome Baker and James L. Quinn, as part of its technical assistance missions in 1967 and 1968, respectively. Their reports to the agency and the Indian government offered a sober assessment of nuclear medicine practice at RMC.

Baker’s report made pointed observations regarding deficiencies in electronics and instrumentation at RMC, noting that he had to spend a considerable portion of his time ‘fixing’ the ‘Phrenicon’ instrument, which had been non-operational for three years. This instrument was vital for both diagnostic applications and research into metabolic reactions and disorders using radioactive compounds. In his report, Baker outlined practical strategies for deploying the Phrenicon more productively and recommended appointing a PhD-trained physiologist to optimise its research potential.^80^ He also emphasised the need for stronger collaboration between universities and RMC to foster metabolic research.^81^ James Quinn’s report similarly offered a comprehensive review. While acknowledging that equipment for multi-probe in-vivo studies, gamma and beta counting, thyroid uptake measurements, radio-respirometry, and tracer biochemistry was ‘quite good’, he found the rectilinear scanners ‘badly outdated’.^82^

Although he praised RMC’s work on malabsorption, anaemia, and retarded growth – conditions commonly present in tropical regions – he noted that such research could be significantly improved ‘with the acquisition of specialised equipment’.^83^ He further observed that the development of new radiopharmaceuticals was hindered by the absence of modern scintigraphic equipment, particularly rapid rectilinear scanners and large stationary imaging devices, both of which were indispensable to first-class nuclear medicine facilities.^84^ Baker also pointed out RMC’s limitation in fully exploiting Technetium- and Indium-labelled compounds, which had become commonplace in advanced countries.^85^ He drew attention to the inadequate coordination between the DAE’s Medical Electronics Group and RMC experts in determining which design features of scanners and related equipment were essential and which were optional.^86^ Quinn, for his part, recommended installing a medical cyclotron to produce short-lived nuclides as part of RMC’s next phase of modernisation.^87^

Quinn’s concluding observation – that nuclear medicine in India lagged ‘disproportionately’ behind global advances – was particularly telling. The absence of a robust domestic radiology industry affected nuclear medicine practice as much as other radiological subfields. The Electronics Corporation of India Limited (ECIL), tasked by the DAE with designing advanced radionuclide scanners, took several years to satisfactorily meet these technical demands. Limitations in electronics and instrumentation thus directly affected the expansion and practice of nuclear medicine in the country. Amidst these technological shortcomings, in 1967, the RMC practitioners established the Society of Nuclear Medicine, India (SNMI) to consolidate nuclear medicine practice and further professional development of their field. The establishment of the SNMI, as distinct from the IRA, was an important milestone in the evolution of Indian radiology, granting nuclear medicine a distinct professional identity.^88^

Concerns, nonetheless, persisted about nuclear medicine’s broader relevance to public health in India. At the society’s annual meeting in November 1975, Bhikaji Gaitonde, Director of the Haffkine Institute in Bombay, urged society members to direct nuclear medicine towards solving ‘problems most pertinent to India’ and promoting a better understanding of tropical diseases.^89^ Warning against reducing nuclear medicine to a mere ‘status symbol’, Gaitonde criticised the blind imitation of Western norms. Indeed, nuclear medicine services had remained concentrated in major urban hospitals rather than extending to district general hospitals.^90^ In December 1976, at a national seminar jointly organised with the World Health Organisation (WHO), the DAE’s Secretary, Homi Sethna, similarly undertook a critical review of the field. Sethna urged the seminar participants to consider, ‘Is it worthwhile to develop nuclear medicine in a country where medical care is in its infancy and public health problems are paramount? … Does it involve spending disproportionately large sums of money for a small return? Does it play any role at all in resolving problems of population, pestilence, and nutrition? … Should there be a small nuclear medicine unit or a large one, depending on the size of the hospital?’^91^ Sethna’s concerns thus reflected an effort to reconcile the promise of nuclear medicine with the realities of scarce resources and pressing public health needs.

Equally compelling was the issue of training and education for current and future nuclear medicine practitioners in the country. Determining the curricular structure for nuclear medicine had to happen amid a prolonged controversy within the IRA over the bifurcation of diagnostic and therapeutic radiology. On the structure of radiological training, which was intensely debated within the IRA, three broad viewpoints were prevalent: a strict separation between the teaching of diagnostic and therapeutic radiology; a combined approach without any separation; and a combined diploma and bifurcation at a higher degree level.^92^ As a result, postgraduate radiological education varied across diploma and degree programs, reflecting what L. R. Parthasarathy, the IRA President of the 1973 Annual Congress, described as a troubling ‘lack of uniformity’ in educational standards. While S. K. Mazumdar initiated the first one-year postgraduate Diploma in Radiation Medicine (DRM) at Delhi University in 1962, the RMC launched its own one-year DRM, affiliated with Bombay University in 1973, which was later extended to two years.^93^

Following nearly a decade of technological challenges, nuclear medicine in India entered a new phase with the arrival of technetium-99m (Tc-99m) radiopharmaceuticals and single-head gamma cameras.^94^ On the one hand, Tc-99m revolutionised diagnostic imaging; on the other hand, the gamma camera significantly enhanced imaging speed and quality, enabling nuclear medicine to move from an experimental, research-oriented activity to a routine clinical service. The installation of a gamma camera at RMC in 1970 enabled rapid imaging of multiple organs, thereby transforming RMC into a comprehensive nuclear medicine centre. In the same year, RMC received the WHO accreditation as a regional centre for thyroid diseases in Asia.^95^

Nevertheless, the acquisition of the latest diagnostic cameras remained constrained by high import costs.^96^ Establishing nuclear medicine departments at all Indian medical colleges and serving their large patient pool necessitated the local design and production of instruments and cameras at affordable prices. It was only in the late 1980s that an indigenously made gamma camera by the Electronics Corporation of India Limited (ECIL), suitable for a range of radiopharmaceuticals, became available, which raised hopes for its rapid dissemination and use.^97^ The adoption of next-generation cameras with tomographic and computerised capabilities was also delayed and did not materialise until the late 1990s and early 2000s.

Amidst such delays and technological struggles, the unveiling of India’s Cobalt-60 teletherapy unit, called Bhabhatron, named after its founding scientist, marked a full circle in India’s aspiration to harness radiotherapy and nuclear medicine. In the 1950s, India received cobalt teletherapy units from Canada when its own atomic program was in infancy. The development of its own Cobalt-60 teletherapy unit therefore was more than just a technological achievement; it reflected a sustained effort to catch up with the West and to provide technical assistance to various least-developed countries as part of its proclaimed anti-colonial solidarity. By the turn of the twenty-first century, the direction of exchange had finally begun to shift. In the 1950s, what had arrived in India as diplomatic aid from the West was now India’s turn to share with other less-developed countries of the world. In doing so, the atomic energy establishment realised yet another postcolonial embodiment: to shift from being a recipient to a provider of technical aid and to extend cancer care beyond the nation.^98^

## Conclusion

In this paper, we set out to map the trajectory of India’s nuclear medicine field through intertwined histories of radiology, cancer care, and atomic energy development. The paper mainly argues that the saga of nuclear medicine in postcolonial India is historically significant for its technopolitical dynamics, highlighting how professional ambitions, state power, and international collaboration intertwined to shape its genesis and evolution. Nuclear medicine, in this sense, was not peripheral to India’s medical history but rather a productive site whereby atomic energy development, in its wake, influenced the modernisation of public healthcare.

The paper also contributes to broader debates on medicine and development by showing how technologically sophisticated medical specialities emerged in developing societies. The radiological community in India had long argued for establishing the field of nuclear medicine, articulating its aspiration for scientific progress and its collective resolve to fight the scourge of cancer. However, in the organisation of nuclear medicine under the auspices of India’s atomic energy program, the technopolitical elites strategically mobilised cancer-cure and healthcare-modernisation narratives, despite the well-known therapeutic benefits and limitations of radiation therapy. It also involved a purposeful appropriation of institutions such as the Tata Memorial Hospital (TMH) in Bombay.

While the postcolonial elites viewed nuclear medicine as vital to disseminating the social benefits of atomic energy, its development within DAE’s biomedical program also served a crucial technopolitical function. It simultaneously advanced the atomic energy programme while expanding specialised cancer care in India. With the concentration of resources and expertise, institutions such as the RMC gradually achieved excellence in research and therapy. At the same time, though, the development of nuclear medicine raised concerns about broader social access and its relevance to public healthcare.

Against the backdrop of persistent inequalities in healthcare access, the emergence of nuclear medicine thus reflected a pattern of technocratic decision-making. It also suggests how the legitimacy of the atomic energy program was built through promises of healthcare modernisation, cancer treatment, and social welfare. Seen in this way, the development of nuclear medicine offers a unique insight into India’s tryst with atomic energy development, along with the lesser-known stories of its biological and medical programs.

The historical account is equally crucial to understanding the zeitgeist of the ‘peaceful atom’ in the early Cold War and the scientific networks that shaped its spread in the developing world. The history of nuclear medicine in India, in this way, reveals a unique biomedical and developmental life of atomic energy, demonstrating how scientific modernity, developmental aspirations, and healthcare provision became deeply intertwined in a postcolonial setting.

## Footnotes

1

Rahul Bhaumik , ‘The History of Colonial Science and Medicine in British India: Centre-Periphery Perspective’, Indian Journal of History of Science, 52, 2 (2017), 174 ; Dhruv Raina , ‘Reconfiguring the Centre: The Structure of Scientific Exchanges Between Colonial India and Europe’, Minerva, 34, 2 (1996), 161–76.

2

Itty Abraham , ‘The Contradictory Spaces of Postcolonial Techno-Science’, Economic and Political Weekly (2006), 210–17; and Pratik Chakrabarti, *Western Science in Modern India: Metropolitan Methods, Colonial Practices* (New Delhi: Permanent Black, 2004).

3 The role of science diplomacy was quite crucial in the development of nuclear medicine across different regions of the world. See John Krige and Kai-Henrik Barth , ‘Introduction: Science, Technology, and International Affairs’, Osiris, 21, 1 (2006), 1–21 ; and Johannes Mattes and Cécile Philippe , ‘Crossing Boundaries, Forging Unity: Nuclear Medicine and Science Diplomacy in Cold War Europe’, The British Journal for the History of Science, 58, 3 (2025), 503–2710.1017/S000708742500031740340850.

4 The arguments about technological lag were particularly pronounced in the healthcare planning discourse after independence, alongside the acute shortage of services, personnel, and equipment. See N. Jivraj Mehta , ‘Inaugural Address Before the Second Indian Congress of Radiology’, Indian Journal of Radiology, 2, 1 (1948), 49–53 .

5 Jawaharlal Nehru, ‘At the ‘Atoms for Health’ Exhibition,’ February 9, 1961, in Madhavan K. Palat (ed.), *Selected Works of Jawaharlal Nehru*, Second Series, vol. 66 (New Delhi: Jawaharlal Nehru Memorial Fund, 2024), 477–79.

6

David Arnold , ‘Nehruvian Science and Postcolonial India’, Isis, 104, 2 (2013), 360–7010.1086/67095423961694.

7 See, Gabrielle Hecht, *The Radiance of France: Nuclear Power and National Identity after World War II*, New edn (Cambridge, MA: MIT Press, 2009); and Gabrielle Hecht (ed.), *Entangled Geographies: Empire and Technopolitics in the Global Cold War* (Cambridge, MA: MIT Press, 2011).

8 Jahnavi Phalkey, in her work, similarly refers to the centralisation of resources for atomic research in postcolonial India, which is relevant for discussion on the organisation of nuclear medicine. See Jahnavi Phalkey, *Atomic State: Big Science in Twentieth-Century India* (Ranikhet: Permanent Black, 2013).

9 A growing body of scholarship in recent years has mapped the emergence of radiology and cancer care in India. Notable among them is Robert D. Smith , ‘The Spread of Radiology in India From 1941 to 1956’, Indian Journal of Cancer, 56, 3 (2019), 285–610.4103/0019-509X.26303931389401; Robert D. Smith , ‘Emerging Infrastructures: The Politics of Radium and the Validation of Radiotherapy in India’s First Tertiary Cancer Hospital’, BioSocieties, 17, 3 (2022), 415–4110.1057/s41292-020-00223-3PMC793360233688371; K. S. Parthasarathy, ‘India’s Tryst With Nuclear Medicine’. *The Write Science*, 25th November 2018; K. S. Parthasarathy , ‘Historical, Regulatory and Safety Aspects of Nuclear Energy in India’, Atoms for Peace: An International Journal, 2, 1 (2008), 102–19; and Itty Abraham, *The Making of the Indian Atomic Bomb: Science, Secrecy and the Postcolonial State* (Hyderabad: Orient Longman, 1998).

10

Robert D. Smith and Mohandas K. Mallath , ‘History of the Growing Burden of Cancer in India: From Antiquity to the 21st Century’, Journal of Global Oncology, 5 (2019), 1–15 10.1200/JGO.19.00048PMC701043631373840. Smith, ‘Emerging Infrastructures’, *op. cit.* (note 9), 11; and Smith, ‘The Spread of Radiology in India From 1941 to 1956’, *op. cit.* (note 9), 285.

11

Venni V. Krishna , ‘The Emergence of the Indian Scientific Community’, Sociological Bulletin, 40, 1–2 (1991), 89–107 .

12 For the elements of postcolonial histories of science, see Arnold, *op. cit.* (note 6), 362.

13
Pratik Chakrabarti, *Medicine and Empire: 1600–1960* (London: Bloomsbury Academic, 2014).

14

Kuppusamy Thayalan , Ramamoorthy Ravicahnadran , and T. S. Swaminathan , ‘First X-Ray Facility in Asia: 125 Years of Excellence’, Indographics, 5, 01 (2026), 001–010 .

15 There were reportedly fewer than 50,000 scientifically trained physicians for the population of over 360,000,000.

16 Unlike popular knowledge, the IRA was originally established on 21st February 1931 by a 10-member group, comprising radiologists from Bombay, Calcutta, and Madras, and held meetings in 1932 and 1938 before becoming inactive during World War II.

17

Rama, P. Rau , ‘Presidential Address Third Indian Congress of Radiology, Calcutta, 25th December 1948’, Indian Journal of Radiology, 3, 1 (1949), 54–61 .

18

M. D. Joshi , ‘Welcome Address on 24th December 1947’, Indian Journal of Radiology, 2, 1 (1948), 48–9.

19

K. Manjunath Rai , ‘Editorial – Our Needs’, Indian Journal of Radiology, 3, 1 (1949), 39–40 .

20

S. N. Deboo , ‘Presidential Address at the Sixth Indian Congress of Radiology’, Indian Journal of Radiology, 6, 3 (1952), 101–5.

21 The founding editors of the journal were Dr Rama Rau, Dr K. Manjunath Rai, and Dr U. Krishna Rau of the Madras branch of the Association.

22 In 1948, the Director General of Health Services had asked IRA to share his views on ‘legislative’ and administrative action to control appropriately the use of radiation for therapeutic and diagnostic purposes’.

23

Indian Radiological Association, ‘Indian Radiological Association Recommendation on Legislative and Administrative Measures for the Control of Practice of Radiology and Purchase of Equipment Act’, Indian Journal of Radiology, 5. 2 (1951), 71–2.

24 The Indian Medical Association (IMA) was one of the largest and most powerful bodies in India’s healthcare ecosystem; therefore, its veto of IRA’s proposal was important.

25 Deboo, *op. cit.* (note 20), 101–5. The IMA’s journal endorsed the IRA’s views on misuse of radiology and held that physicians and surgeons already possessing X-ray units should either complete refresher courses or provide evidence of their knowledge of the hazards of ionising radiation and their competence to adopt necessary protective measures.

26 For more discussion on cancer incidence in India, see Smith and Mallath, ‘History of the Growing Burden of Cancer in India’, *op. cit.* (note 10), 1–15.

27

V. R. Khanolkar , ‘Cancer in India – Lecture Delivered at Lady Hardinge Medical College, New Delhi, 20th March 1950’, Vijnan-Karmee, 2, 5 (1950), 4–5 .

28 Deboo, *op. cit.* (note 20), 101–5.

29

N. B. Roy , ‘Presidential Address at the Fourteenth Congress of Radiology, Hyderabad, 1960’, Indian Journal of Radiology, 13, 1 (1961), 197–204 .

30 Mehta, ‘Inaugural Address Before the Second Indian Congress of Radiology’, *op. cit.* (note 4), 49–53.

31
*Ibid.*, 51.

32

Susovan Banerjee , Umesh Mahantshetty , and Shyamkishore Shrivastava , ‘Brachytherapy in India–a Long Road Ahead’, Journal of Contemporary Brachytherapy, 6, 3 (2014), 331–510.5114/jcb.2014.45761PMC420019025337139.

33

K. P. Mody , ‘Presidential Address by Dr K.P. Mody, Head of the Department of Radiology, Tata Memorial Hospital, Bombay’, Indian Journal of Radiology, 2, 1 (1948), 53–6.

34 The society was established under the patronage of Mr Naval Hormusji Tata, a noted industrialist and philanthropist from the Tata Group, and was affiliated with the International Union against Cancer in Paris. It was led by noted Indian pathologist, Dr V. R. Khanolkar from Tata Memorial Hospital.

35

N. B. Roy , ‘Radiotherapy Organisation – Paper Read at the 8th Indian Congress of Radiology, Madras, 1954’, Indian Journal of Radiology, 8, 4 (1954), 192–8; and N. B. Roy , ‘Recent Advances in Radiotherapy – Paper Read at the 5th Indian Congress of Radiology, Calcutta, 1951’, Indian Journal of Radiology, 5, 1 (1951), 16–25 .

36
*Ibid.* Roy, ‘Radiotherapy Organisation’, 198. “Heart within and God overhead” served as a rallying metaphor for India’s radiological elites, invoking both the determination to build centralised radiotherapy institutions and the collective resolve to confront cancer disease through coordinated efforts.

37

K. Manjunath Rai , ‘Editorial’, Indian Journal of Radiology, 2, 2 (1948), 103–4.

38

R. F. Sethna , ‘Presidential Address’, Indian Journal of Radiology, 13, 1 (1959), 39–49 .

39 Radioisotopes are the unstable form of an element that emits radiation to transform into a more stable form. Due to their ability to emit radiation and induce changes in living materials, they can act as tracers, provide radiation for treatment and sterilisation, help improve agricultural production, assist industrial testing, and support scientific research. See Jerry M. Obaldo and Barbara E. Hertz , ‘The Early Years of Nuclear Medicine: A Retelling’, Asia Oceania Journal of Nuclear Medicine and Biology, 9, 2 (2021), 207; and “Isotopes in Medical Diagnosis: New Techniques Reviewed at Vienna Symposium,” *IAEA Bulletin*, 1, 1 (April 1959), 3–5.10.22038/aojnmb.2021.55514.1385PMC825551934250151

40 Néstor Herran and Sebastian Grevsmühl, ‘The First Nuclear Industry: Radioisotopes, State and Society’. PhD diss., CNRS; UPMC; EHESS, 2017.

41 ‘Atomic Medicine-New World-Wide Science’, *The Hindustan Times*, 05 December 1948. Angela N. H. Creager has critically examined the use of radioisotopes as political instruments by the U.S. during the early Cold War years. See, Angela N. H. Creager , ‘Radioisotopes as Political Instruments, 1946–1953’, Dynamis, 29 (2009), 219–3910.4321/s0211-95362009000100010PMC292345420725612; and Angela N. H. Creager, *Life Atomic: A History of Radioisotopes in Science and Medicine* (Chicago: University of Chicago Press, 2013).

42

Sten Carlson , ‘A Glance at the History of Nuclear Medicine’, Acta Oncologica, 34, 8 (1995), 1095–10210.3109/028418695091272368608034.

43 K. P. Mody, ‘Speech by Dr K.P. Mody, Chairman of the Reception Committee at the Sixth Indian Congress of Radiology, 8–11th February 1952’, *TIFR Archives*, D-2004-00013-17(1-24).

44 On the use of radium in therapy, see Charles Hayter, *Element of Hope: Radium and the Response to Cancer in Canada, 1900–1940* (McGill-Queen’s Press-MQUP, 2005). Also see John Krige , ‘The Politics of Phosphorus-32: A Cold War Fable Based on Fact’, Historical Studies in the Physical and Biological Sciences, 36 (2005), 71–91 .

45 ‘Radioactive Medicine Used in Calcutta: Treatment of Cancer May Be Attempted Shortly’. *The Times of India (Calcutta)*, 31 October 1952. The U.S. Atomic Energy Commission also sent a shipment of radio cobalt to the Department of Scientific and Industrial Research (DSIR) in India for research purposes in September 1950; see United States Atomic Energy Commission (USAEC), ‘Monthly Report of Foreign Shipments: Radioisotopes – Shipment 920, Cobalt-60 to the Department of Scientific and Industrial Research, New Delhi’. Report No. 37, U.S AEC. *National Archives and Records Administration.* USA, September 1950. The author gratefully acknowledges receiving this report from Angela N. H. Creager.

46 ‘Letter Addressed to Col. P. B. Bharucha From Dr D. J. Jussawalla’. *Tata Memorial Hospital.* 3rd October 1955.

47

A. N. K. Menon , ‘Editorial – Radioisotopes in Medicine’, Indian Journal of Radiology, 15, 2 (1961), i–iii . For uses of isotopes in agriculture, see Kapil Patil , ‘In Pursuit of “Man-Made Evolution” Peaceful Atoms, Circulatory Knowledge, and Atomic Agriculture in India’, Agricultural History, 98, 4 (2024), 674–701 .

48 Roy, *op. cit.* (note 29), 197–204.

49 See Phalkey, *op. cit.* (note 8).

50 For the life and works of Bhabha, see William George Penney, “Homi Jehangir Bhabha, 1909–1966,” *Biographical Memoirs of Fellows of the Royal Society*, 13 (1967), 35–55; and Indira Chowdhury and Ananya Dasgupta, *A Masterful Spirit: Homi J. Bhabha, 1909–1966* (New Delhi: Penguin Books, 2010).

51

K. M. Rai , ‘The 12th Indian Congress of Radiology – Editorial Comment’, Indian Journal of Radiology, 12, 1 (1958), 1–4 .

52 Phalkey, *op. cit.* (note 8).

53 Deepak Kumar, “Emergence of ‘Scientocracy’: Snippets from Colonial India,” *Economic and Political Weekly*, 39, 35 (2004), 3893–98.

54

A. Nagaratnam , ‘Activities and Achievements of INMAS’, Defence Science Journal, 40, 4 (1990), 325–34. The radiation cell was originally set up by Daulat Singh Kothari, who served as a scientific advisor to India’s defence ministry and led the study on the biological effects of ionising radiation. In 1959, Mazumdar had reportedly investigated 59 patients with Iodine-131 isotopes and ‘treated ten cases of thyrotoxicosis’, marking one of the earliest clinical uses of radioiodine in India.

55 The ‘Atoms for Peace’ was announced by President Eisenhower at the United Nations in December 1953. For its Cold War underpinnings and polyvalent motivations, see John Krige , ‘Atoms for Peace, Scientific Internationalism, and Scientific Intelligence’, Osiris, 21, 1 (2006), 161–81.

56 Colombo Plan was a shorthand for the Colombo Plan for Co-operative Economic Development in South and Southeast Asia. As McGee and Manson have noted, through the Colombo Plan, the Anglosphere countries sought to integrate their Southeast Asian counterparts into the project of Western Cold War globalisation, with Canada playing a central role. See ‘Inauguration of Atomic Reactor: Atomic Power for Nation’s Uplift: Nehru Hails Help of friendly Countries’. *The Times of India* (Mumbai). 17 January 1961; David McGee and Rian Manson, “Canada, Communism, and the Colombo Plan,” in Nina Möllers and Bryan Dewalt (eds), *Objects in Motion: Globalizing Technology* (Washington, DC: Smithsonian Institution Scholarly Press, 2016) 46–64; and David C. Engerman, *The Price of Aid: The Economic Cold War in India* (Cambridge, MA: Harvard University Press, 2018).

57 The reactor was named CIRUS, which stood for Canada-India-Reactor-US. The US contributed to the project by supplying heavy water for the reactor. See A. S. Godinho, ‘Atomic Age Dawns in Our Country’, *The Times of India.* 6 May 1956; and ‘Inauguration of Atomic Reactor: Atomic Power for Nation’s Uplift: Nehru Hails Help of Friendly Countries’, *The Times of India* (Mumbai), India. 17 January 1961.

58

V. Iya and R. Mani , ‘Radiopharmaceuticals Pattern of Development and Utilisation in India’, Defence Science Journal, 40, 4 (2013), 351–6.

59 Nehru, *op. cit.* (note 5), 477.

60
*Ibid.*

61 Naushir Bharucha was an independent Member of Lok Sabha (the lower house of the Parliament) from East Khandesh constituency, Bombay, 1957–62.

62 Bharucha, Naushir. “Interventions during the Debate on the Cut Motions on the Demands for Grants for the Department of Atomic Energy.” *Lok Sabha Debates*, Second Series, vol. XXVII, March 16, 1959, cols. 6383–6393. New Delhi: Lok Sabha Secretariat.

63
*Ibid.*

64 Jawaharlal Nehru, “Interventions during the Debate on the Cut Motions on the Demands for Grants for the Department of Atomic Energy,” in *Lok Sabha Debates*, Second Series, vol. XXVII, March 16, cols. 6415–6426 (New Delhi: Lok Sabha Secretariat, 1959).

65

Soraya Boudia , ‘Radioisotopes “Economy of Promises”: On the Limits of Biomedicine in Public Legitimization of Nuclear Activities’, Dynamis 29 (2009), 241–259 10.4321/s0211-9536200900010001119852394.

66

Nishu Singh Goel , *Indelible Footprints on the Sands of Time*: *Commemorating Seventy-Five Years of Tata Memorial Centre.* Mumbai: Tata Memorial Center, 2017 .

67 Boudia, *op. cit.* (note 65), 244–5. For the history of USAEC, see Richard Hewlett , Oscar Anderson , and Francis Duncan , A History of the United States Atomic Energy Commission, 2 vols. Berkeley: University of California Press, 1990 .

68 Jawaharlal Nehru, ‘For Vishnu Sahay: Cancer Research at Atomic Energy Department’, 12 December 1961, *Selected Works of Jawaharlal Nehru*, Second Series, Vol. 73, 481.

69
*Ibid.*

70

BARC, Radiation Medicine Centre – Silver Jubilee 1963–1988 (Mumbai: Bhabha Atomic Research Centre, May 1989).

71 “India—Report and Memorandum.” 1961. Folder 56, *John Hundale Lawrence Papers, 1935–1987* (BANC MSS 87/86 c). The Bancroft Library, University of California, Berkeley.

72 Government of India, Department of Atomic Energy, *Annual Report of the Department of Atomic Energy for the Year 1962–63* (New Delhi: Government of India, 1963), 10.

73 BARC, *op. cit.* (note 70).

74 RMC, *Memoirs: Radiation Medicine Centre-Golden Jubilee 1963–2013*, Department of Atomic Energy (Mumbai: Government of India, 2013).

75

Kenji Ito and Maria Rentetzi , ‘The Co-Production of Nuclear Science and Diplomacy: Towards a Transnational Understanding of Nuclear Things’, History and Technology, 37, 1 (2021), 4–20 .

76 Government of India, Department of Atomic Energy, *Annual Report of the Department of Atomic Energy for the Year 1963–64* (New Delhi: Government of India, 1964), 19.

77 Nagaratnam, *op. cit.* (note 54), 325–34.

78 R. D. Ganatra, S. M. Sharma, et al. ‘Radioisotopic Investigations of Some Tropical Diseases’. In *Peaceful Uses of Atomic Energy. Proceedings of the Fourth International Conference*, jointly sponsored by the UN and the IAEA and held in Geneva, 6–16 September 1971. Vol. 13. 1972.

79 The RMC had received the initial instruments set from Nobel Laureate Ernest O. Lawrence, the cyclotron inventor. See Department of Atomic Energy, “Chapter 2: Radioisotopes and Radiation in Health Sector: Ensuring Better Healthcare for the Nation,” in *Saga of Atomic Energy in India: Golden Jubilee Commemorative, Vol. 3* (Mumbai: Department of Atomic Energy, 2005), 24. Also, for the history of the slow-rectilinear scanner, see William J. MacIntyre and C. Craig Harris , ‘The Decline and Fall of the Rectilinear Scanner: Nuclear Medicine Instrumentation 1970–1995’, Journal of Nuclear Medicine Technology, 23, 4 suppl (1995), 16S–20S .

80 Nome Baker, ‘Radioisotope Techniques in Breath Analysis – Report to the Government of India, October–December 1967’, *International Atomic Energy Agency*, TA Report 379, WP/5/379, 9th May 1968.

81
*Ibid.*, 05.

82 J. L. Quinn, ‘Medical Applications of Radioisotopes – Report to the Government of India, October–November 1968’, *International Atomic Energy Agency*, TA Report 428, WP/5/428, 14th April 1969, 1–12.

83
*Ibid.*, 2.

84
*Ibid.*

85
*Ibid.*, 3.

86
*Ibid.*, 4–5.

87
*Ibid.*, 10.

88 The society held its first annual meeting at RMC on 25th August 1968.

89 Bhikaji Gaitonde, Director of Haffkins Institute, addressed the seventh annual conference of SNMI held in Calcutta, as its president. See Walter Seelantag, ‘Letter From Walter Seelantag’. *World Health Organisation* (WHO). No. 157/3304/70. World Health Organiation (WHO) Archives. Also, see Ramniklal, D. Ganatra, ‘WHO Circular Letter of Nuclear Medicine No. 2’. *WHO Nuclear Medicine Network*, A14/286/6, World Health Organisation (WHO) Archives.

90 According to AERB statistics for 2018, only 14 per cent of licensed nuclear medicine facilities were government-owned, and the remaining 86 per cent were privately owned.

91 Bhabha Atomic Research Centre, Radiation Medicine Centre *Report on the National Seminar in Nuclear Medicine* (Bombay: Department of Atomic Energy, 1979). Available through the IAEA International Nuclear Information System (INIS).

92 In a debate on radiological education, successive IRA presidents held different views in their Presidential Addresses, which reflected the state of radiological education in India. These included Dr. L. H. Athele (Presidential Address, 1961), Dr A. R. Deshpande (Presidential Address, 1967), Dr Yusuf Siddique (Presidential Address, 1968), and Dr L. R. Parthasarathy (Presidential Address, 1973).

93

Anshu Rajnish Sharma , ‘Nuclear Medicine in India: A Historical Journey’, Indian Journal of Nuclear Medicine, 33, Suppl 1 (2018), S5–S10 10.4103/0972-3919.245053PMC624372130533977. At present, the DRM courses are governed by the DAE-led Homi Bhabha National Institute (HBNI), Bombay.

94 N. Ramamoorthy, “Foreword,” in *RMC Golden Jubilee, 1963–2013* (Mumbai: Radiation Medicine Centre, Bhabha Atomic Research Centre, 2013), 10–11. Also see, V. Iya and R. Mani , ‘Radiopharmaceuticals: Pattern of Development and Utilisation in India’, Defence Science Journal, 40, 4 (1990), 351–6.

95 Letter from Walter Seelantag, WHO. In 1969, the IAEA and the WHO designated the RMC, Southeast Asia’s Regional Reference Centre for Nuclear Medicine. No. 157/3304/70. WHO Archives.

96
*op. cit.* (note 94).

97

Ramachandra Datatraya Lele , ‘Nuclear Medicine in India’, Defence Science Journal, 40, 4 (October 1990), 347–50.

98 Press Trust of India, “Narendra Modi Hands Over Bhabhatron to Mongolia for Cancer Treatment,” *Business Standard,* May 17, 2015, https://www.business-standard.com/article/pti-stories/modi-hands-over-bhabhatron-to-mongolia-for-cancer-treatment-115051700356_1.html.

## Archives Consulted

Tata Institute of Fundamental Research, Mumbai, India.

Tata Central Archives, Pune.

National Medical Library, New Delhi.

Prime Minister’s Memorial and Museum Library, New Delhi.

International Atomic Energy Agency (IAEA) Archives, Vienna.

World Health Organization (WHO) Archives, Geneva.

